# MicroRNA-362-3p Inhibits Migration and Invasion via Targeting BCAP31 in Cervical Cancer

**DOI:** 10.3389/fmolb.2020.00107

**Published:** 2020-06-09

**Authors:** Shuya Yang, Xiyang Zhang, Yuanjie Sun, Jingqi Shi, Dongbo Jiang, Jing Wang, Yang Liu, Chenchen Hu, Jingyu Pan, Lianhe Zheng, Kun Yang

**Affiliations:** ^1^Department of Immunology, The Fourth Military Medical University, Xi'an, China; ^2^Department of Orthopedics, Tangdu Hospital, The Fourth Military Medical University, Xi'an, China

**Keywords:** miR-362-3p, BCAP31, cervical cancer, migration, invasion

## Abstract

Cervical cancer (CC) is the most common malignant tumor in gynecology, and metastasis is an important cause of patient death. MiRNAs (microRNAs) have been found to play key roles in cervical cancer metastasis, but the effect of miR-362-3p in CC is unclear. This study aimed to investigate the role of miR-362-3p in cervical cancer migration and invasion. We compared the expression levels of miR-362-3p in cervical cancer tissues and adjacent normal cervical tissues. In CC tissues, miR-362-3p expression was significantly down-regulated, which is related to the cancer stage and patient survival. MiR-362-3p can effectively inhibit the migration and invasion of cervical cancer cells. The dual-luciferase reporter assay results showed that BCAP31 (B cell receptor associated protein 31) is a direct target protein of miR-362-3p. The results of the immunohistochemical examination of clinical tissue samples showed that BCAP31 was abnormally highly expressed in cervical cancer, which was positively correlated with the clinical stage. BCAP31 knockdown exerted similar effects as miR-362-3p overexpression. Further GSEA analysis showed that BCAP31 may participate in multiple biological processes, such as protein transport, metabolism, and organelle organization. Our results suggest that miR-362-3p inhibits migration and invasion via directly targeting BCAP31 in cervical cancer, and restoring miR-362-3p levels may be a new treatment strategy for cervical cancer in the future.

## Introduction

Cervical cancer (CC) is one of the common malignant tumors in women's reproductive systems and the third leading cause of cancer mortality among females (Torre et al., 2015; Small et al., 2017). Local recurrence and distant metastasis are major causes of death. Despite advances in surgery combined with radiotherapy and/or chemotherapy, some CC patients with early metastases result in poor prognosis and therapeutic effect (Wright et al., 2015; Guo et al., 2018; Nanthamongkolkul and Hanprasertpong, 2018; Xu et al., 2018). Therefore, research on the molecular mechanism that promotes CC metastases is of great significance.

MicroRNA (miRNA) is a type of non-coding small RNA found in animals, plants and some viruses, which plays an important role in the post-transcriptional regulation of gene expression (O'Brien et al., 2018). Abnormal changes in miRNA expression have been reported in several human cancers and are related to tumorigenesis and development (Reddy, 2015; Peng and Croce, 2016). It has been reported that some miRNAs, such as miR-1246, miR-221-3p, miR-20a, and miR-92a, etc., are involved in the regulation of CC invasion and metastasis (Kang et al., 2012; Chen et al., 2014; Zhou et al., 2015; Wei et al., 2017). However, the miRNA mechanism of cervical cancer metastasis has not been fully elucidated.

MiR-362-3p is downregulated in cervical cancer and acts as an oncosuppressor miRNA. MiR-362-3p can participate in regulating cell proliferation through MCM5, and is positively correlated with the survival time of CC patients (Wang et al., 2018; Song et al., 2019). However, its effect in CC metastasis is not clear. In our research, we focused on the role of miR-362-3p in cervical cancer metastasis. We analyzed the correlation between miR-362-3p expression level and prognosis in patients with CC, and explored the role of miR-362-3p in the regulation of invasion and migration of cervical cancer cells. Our results show for the first time that miR-362-3p regulates the invasion and migration of cervical cancer cells by regulating BCAP31 as a direct target gene. Collectively, our findings suggest that miR-362-3p is a promising target for CC treatment and a potential prognostic marker in CC.

## Materials and Methods

### Clinical Tissue Sample Collection

Primary cervical carcinomas and adjacent normal cervical tissues were obtained from patients with clinically and pathologically confirmed cervical carcinomas at Xijing Hospital, the Fourth Military and Medical University (FMMU, Xi'an, China). All samples were collected with informed consent from all subjects. The study was approved by the Medical Ethics Committee of FMMU, Xi'an, China.

### Cell Culture and Transfection

The cell lines (Genechem Co., Ltd., Shanghai, China) were cultured at 37°C with 5% CO_2_ in Dulbecco's modified Eagle's medium (Gibco) and minimum essential media (Gibco), respectively, supplemented with 10% fetal bovine serum. MiR-362-3p mimics, negative control and BCAP31 siRNA were transiently transfected using the Lipofectamine 3000 reagent according to the manufacturer's instructions. The miRNA mimics, negative controls and BAP31 siRNA were synthesized by GenePharma and Sangon Biotech. The sequences were as follows: 5′-UUCUCCGAACGUGUCACGUTT-3′(sense) and 5′-ACGUGACACGUUCGGAGAATT-3′(antisense) for negative control, 5′-AACACACCUAUUCAAGGAUUCA-3′(sense) and 5′-AAUCCUUGAAUAGGUGUGUUUU-3′(antisense) for miR-362-3p mimics, 5′-GGUGAACCUCCAGAACAAUTT-3′(sense), and 5′-AUUGUUCUGGAGGUUCACCTT-3′ (antisense) for BCAP31 siRNA.

### Quantitative Real-Time PCR Analysis (qRT-PCR)

Total RNA from cells or tissues was extracted using the TRIzol reagent (Takara) according to the manufacturer's instructions. Reverse transcription reactions for miRNAs were performed with SYBR^®^ PrimeScript™ miRNA RT-PCR Kit (TaKaRa Bio Group, Shiga, Japan). U6 RNA was used as the internal control. All samples were normalized to the internal controls, and fold changes were calculated via the relative quantification method (2^−ΔΔCT^). The primers were as follows: 5′-GCCGAAACACACCTATTCAAG-3′(forward) and 5′-TATGGTTTTGACGACTGTGTGAT-3′(reverse) for miR-362-3p, 5′-ATTGGAACGATACAGAGAAGATT-3′(forward) and 5′-GGAACGCTTCACGAATTTG-3′(reverse) for U6.

### Wound Healing Assay

Transfected cancer cells were cultured into six-well cell plates. When the cell confluence reached 90%, mitomycin C was added to inhibit cell proliferation for 1 h, and then directly scraped with a pipette tip. Images were captured continuously at 0, 24, and 48 h. All experiments were repeated at least three times.

### Migration and Invasion Assay

The migration and invasion abilities of HeLa and SiHa cells were detected by the Transwell assay. Transwell chamber with or without matrigel-coated membrane for detection of invasion and migration, respectively. Cells were digested to get cell suspensions after cell transfection and mitomycin C treatment. The cell suspension was placed into the upper chamber in 600 μL of serum-free Dulbecco's modified Eagle's medium (DMEM). DMEM medium supplemented with 10% FBS was placed in the lower chamber as a chemoattractant. After the cells were cultured for 48 h, the cells were fixed with 4% paraformaldehyde, stained with crystal violet, imaged and counted. All experiments were repeated at least three times.

HoloMonitor M4 was also used for cell migration ability test. According to the instructions, plant the transfected cells into the plate. Five cells were randomly selected in the field of view, monitored for 96 h. The cell migration route was drawn to calculate the cell migration distance.

### xCELLigence Real-Time Cellular Analysis (RTCA)

Real-time cell migration was measured using the xCELLigence RTCA DP instrument equipped with a CIM-plate 16. The CIM-plate 16 is a 16-well system in which each well is composed of upper and lower chambers separated by an 8-μm microporous membrane. The cell suspension was placed into the upper chamber in serum-free DMEM. DMEM medium supplemented with 10% FBS was placed in the bottom chamber as a chemoattractant. Migration is measured as the relative rate of change (cell index) across microelectronics integrated into the bottom side of the membrane. Cells were monitored and expressed as a cell index value (CI) every 15 min. The cell index indicating the time point is averaged from at least three independent measurements. All data were recorded using RTCA software.

### Dual-Luciferase Reporter Assay

Human BCAP31 3′-UTR (3′-untranslated region) reporter plasmids containing the putative binding sequence of miR-362-3p (wild-type, WT) and its mutant sequence (mutant, MUT) were amplified by qRT-PCR, inserted into the GP-miRGLO reporter vector (GenePharma, Shanghai, China), and validated by sequencing. HeLa and SiHa cells were plated in a 48-well plate and cotransfected with BCAP31 3′-UTR WT/MUT vectors and negative control (NC)/miR-362-3p mimics. Luciferase activity was measured 48 h after transfection using the Dual-Luciferase Reporter Assay System (Promega, Wisconsin, USA).

### Western Blotting Analysis

The western blotting analysis was used to detect protein BCAP31 and β-actin expression. RIPA cell lysate containing protease inhibitor was used to lyse the cells for 30 min. After centrifugation at 12,000 g for 15 min at 4°C, the supernatant was obtained. After electrophoresis, the protein sample was transferred to a PVDF membrane, and the 5% skim milk powder was sealed at room temperature for 1 h. Rabbit anti-human BCAP31 antibody (Abcam, Cambridge, MA) or mouse anti-human Actin antibody (Proteintech, Chicago, USA) were added separately and incubated overnight at 4°C. After washing with TBST, membranes were incubated with secondary antibody at room temperature for 2 h. TBST was used to clean the membrane 3 times. Bands were detected using the ECL Kit and β-Actin was used as a loading control. Repeat the experiment at least three times.

### Immunohistochemistry Staining

Approximately 4-μm thick paraffin sections were stained with hematoxylin-eosin (HE) stain and anti-BAP31 antibodies. Sections were blocked with 3% H_2_O_2_ to eliminate endogenous tissue peroxidase followed by heat-mediated antigen retrieval with Tris/EDTA (pH 9.0) buffer. The specimens were incubated with anti-BAP31 antibodies overnight at 4°C. HRP-labeled secondary antibodies were incubated with the sections for 1 h at room temperature before immunostaining was performed with DAB chromogen (Dako). The immunohistochemical staining was digitalized with the Nanozoomer Digital Pathology System (Hamamatsu, Herrsching, Germany). Specimens were reviewed for staining intensity and staining extent.

### Bioinformatics Analysis

The RNA-seq data of patients and their corresponding clinical information were downloaded from TCGA (The Cancer Genome Atlas) database (https://xenabrowser.net/datapages/). The high and low expression of miR-362-3p is divided into two groups based on the median. The survival analyses of miR-362-3p in cancers were performed with the R language (Ribobio, Guangzhou, China). The GSEA (Gene set enrichment analysis) was performed on the expression data of 303 cervical cancer patients performed with the R language. Nominal *p*-value was used to estimate the statistical significance of the enrichment score.

### Statistical Analysis

All the experiments were repeated at least three times, and statistical analyses were performed using GraphPad Prism 7.0 (GraphPad Software, San Diego, CA). For experiments with more than two groups, the differences between groups were compared by one-way ANOVA and the Dunnett test, in which all groups were tested with the control group as a reference. For experiments with only two groups, Student's *t*-test was used for comparisons of group means. Survival rates were analyzed by the Kaplan-Meier method. A Chi-square test was used to evaluate the correlation of clinicopathologic features between two groups with differential expression. The Spearman test was applied to explore the correlation between BCAP31 expression and miR-362-3p expression. *P* < 0.05 was considered to be statistically significant.

## Results

### MiR-362-3p Is Underexpressed in Cervical Cancer Tissues and Correlated With Patient Survival

**Figure 1:**
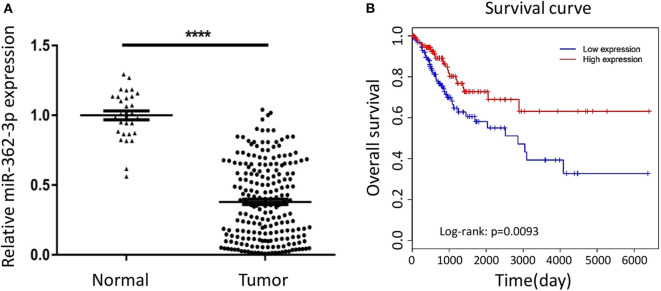
MiR-362-3p is lowly expressed in cervical cancer tissues and correlated with patient survival. **(A)** Expression of miR-362-3p in 208 human cervical cancer tissues and 30 normal adjacent tissues. Datas are the relative expression normalized to U6. ^****^P < 0.0001. **(B)** Kaplan–Meier survival curve showing overall survival of cervical cancer patients with low or high levels of miR-362-3p expression from TCGA database.

To explore the expression characteristics of miR-362-3p in cervical cancer, we performed a real-time quantitative PCR experiment to compare the expression of miR-362-3p in cervical cancer tissues (*n* = 208) and adjacent normal tissues (*n* = 30). The results showed that miR-362-3p expression in cervical cancer was about 35.7% of normal tissues (Figure 1A).

In order to further evaluate the clinical significance of miR-362-3p, we analyzed the relationship between miR-362-3p expression levels and some factors of CC patients, such as age, tumor size, lymph node, grade, and tumor stage. We divided patients into high/low expression groups based on the median expression level of miR-362-3p. The correlation between miRNA and each factor was compared, and the results showed that miR-362-3p expression was related to the patient's grade and tumor stage (Table 1).

**Table 1:** Correlation between expression of miR-362 in cancer cell and clinicopathological parameters.

**Factor**	**Low miR-362**		**High miR-362**		***P*-value**
	**No**.	**%**	**No**.	**%**	
All patients	104	50.0	104	50.0	
Age (years)					
≤ 40	20	9.6	25	12.0	
>40	84	40.4	79	38.0	0.400
Tumor size					
T1	65	31.25	67	32.2	
T2 or T3	39	18.75	37	17.8	0.773
Lymph node					
N0	97	46.6	102	49.0	
N1	7	3.4	2	1.0	0.088
Grade					
–	6	2.9	8	3.8	
1	7	3.4	24	11.5	
2 or 3	91	43.8	72	34.6	**0.0007**
Stage					
I or II	95	45.7	102	49.0	
III or IV	9	4.3	2	1.0	**0.030**

*No. means the number of cases. Bold values indicates that the p < 0.05, which represents a statistical difference*.

To further verify the correlation between miR-362-3p expression level and patient prognosis, we analyzed the relationship between high/low expression of miR-362-3p and CC patient survival from the TCGA (The Cancer Genome Atlas) database. The results showed that patients with high miR-362-3p expression had longer survival time in nine kinds of cancers, including CC (Figure 1B and Supplementary Figure S1). These results suggest that miR-362-3p may play an important role in the development of cervical cancer (Figure 1B).

### MiR-362-3p Inhibits Migration and Invasion of Cervical Cancer Cells

**Figure 2:**
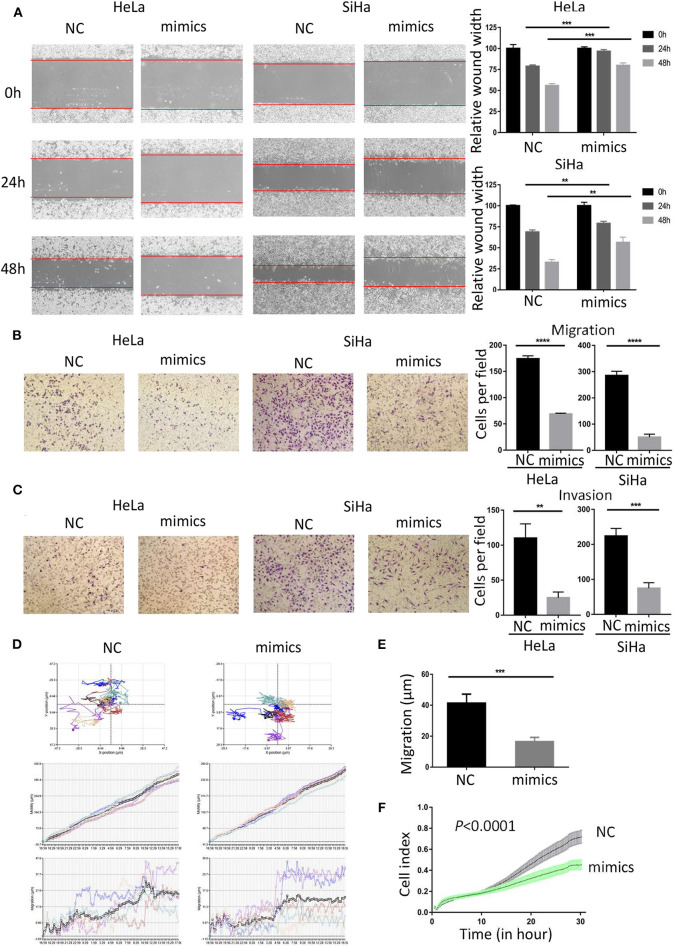
MiR-362-3p inhibits migration and invasion of cervical cancer cells. **(A)** Wound-healing experiments showed the migration of HeLa and SiHa cells transfected with negative control (NC) or miR-362-3p mimics. **(B)** Transwell migration assay of HeLa and SiHa cells after transfected with NC or miR-362-3p mimics. **(C)** Transwell invasion assay of HeLa and SiHa cells treated as in **(B)**. **(D)** HeLa cell movement tracks were recorded by HoloMonitor M4 to show cell migration ability, after transfected with NC or miR-362-3p mimics. **(E)** Cell migration distance results from five cells randomly selected in different directions after transfected with NC or miR-362-3p mimics in HeLa cells, tested by HoloMonitor M4. **(F)** Real-time cell migration was measured using the xCELLigence RTCA DP system, for HeLa cells transfected with NC or miR-362-3p mimics. ^**^P < 0.01; ^***^P < 0.001; ^****^P < 0.0001. Datas are represented as the mean ± s.d. of three independent experiments.

To investigate the role of miR-362-3p in CC cells, we transiently transfected cervical cancer HeLa and SiHa cells with negative control/miR-362-3p mimics. The wound healing assay results showed that the mimics-transfected cells had a weaker healing ability, compared with the control group (Figure 2A). The results were consistent in SiHa cells. The effect of miR-362-3p mimics on migration and invasion of cervical cancer cells was further tested by transwell experiments. The results showed that there was no significant difference in cell counts between the two groups after mitomycin C treatment (Supplementary Figure S2). The cell migration and invasion of the transfection group were 39.6 and 22.7% of that in the control group in HeLa cells, similarly, 17.7 and 33.4% in SiHa cells (Figures 2B,C).

HoloMonitor M4 was also used for cell migration ability test. The cell migration trajectory was continuously tracked and recorded for 96 h. The results of cell trajectory and migration distance revealed that the cell motility of the transfected group was weaker in HeLa (Figures 2D,E). We further used the xCELLigence RTCA DP system to analyze the differences in cell migration capacity between the two groups. The results of RTCA showed that the number of HeLa cell migration in the transfected group was less than that in the control group (Figure 2F).

The above results indicate that miR-362-3p plays a role in cervical cancer migration and invasion. Increasing the expression level of miR-362-3p can effectively inhibit the cell's metastatic ability.

### BCAP31 Is a Direct Target of miR-362-3p in Cervical Cancer

**Figure 3:**
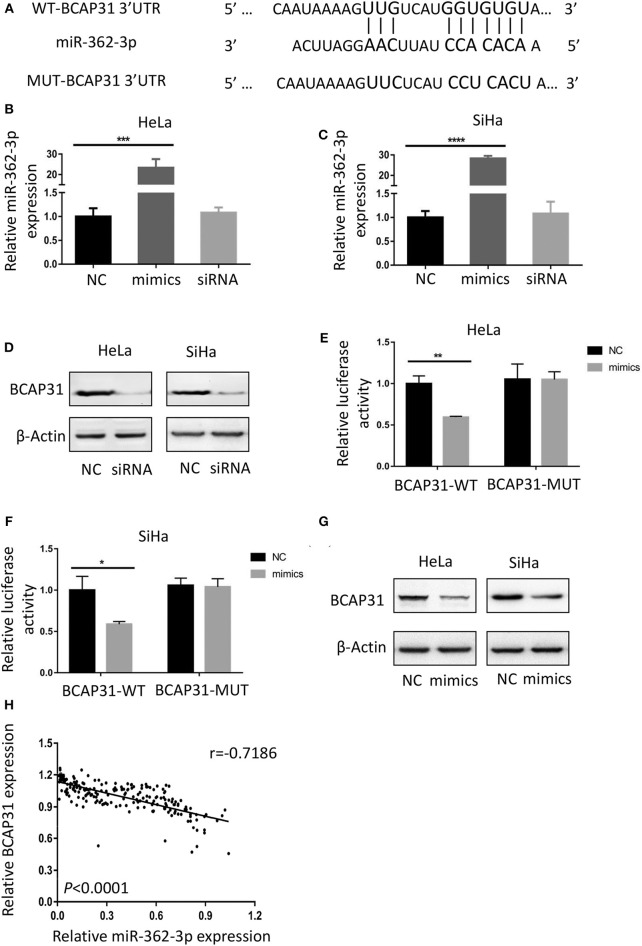
BCAP31 is a direct target of miR-362-3p in cervical cancer. **(A)** Complementarity of the 3′-UTR of wild-type (WT) or mutant (MUT) human BCAP31 mRNA (starting at nucleotide +417) with the miR-362-3p seed sequence. **(B,C)** qRT-PCR analyses for miR-362-3p expression following transfection of miR-362 mimics, NC (negative control) and siRNA of BAP31 into HeLa of SiHa cells, U6 RNA was used as control. **(D)** Western blotting results of BCAP31 expression following transfection of NC (negative control) and siRNA into HeLa or SiHa cells, β-Actin was used as control. **(E,F)** Relative luciferase activity in HeLa and SiHa cell line co-transfected with negative control/miR-362-3p mimics and BCAP31-WT/MUT vector. **(G)** Western blotting results of BCAP31 expression following transfection of NC (negative control) and miR-362-3p mimics into HeLa or SiHa cells, β-Actin was used as control. **(H)** An inverse correlation between miR-362-3p and BCAP31 expression in cervical cancer tissues. ^*^P < 0.05; ^**^P < 0.01; ^***^P < 0.001; ^****^P < 0.0001. Datas are represented as the mean ± s.d. of three independent experiments.

To identify the potential target of miR-362-3p, we used the target prediction websites TargetScan and miRNA.org, and focused on the BCAP31 gene. The websites predicted that the 3′-UTR of BCAP31 mRNA contains a complementary sequence for the seed region of miR-362-3p. Based on this, we constructed the wild type and mutant plasmids of BCAP31 3′-UTR (Figure 3A).

We used real-time quantitative PCR to detect changes in miR-362-3p expression after transfection of the negative control/miR-362-3p mimics/BCAP31 siRNA into HeLa or SiHa cells. The results showed that miR-362-3p mimics could effectively increase miR-362-3p expression, while BCAP31 siRNA did not alter miR-362-3p expression level (Figures 3B,C). Western blotting results showed that BCAP31 siRNA could significantly reduce BCAP31 protein expression in cells (Figure 3D).

In the Dual-luciferase reporter assay, the cotransfection of miR-362-3p mimics significantly decreased the WT BCAP31 luciferase reporter activity, while the luciferase activity was not reduced when the cells were transfected with the MUT BCAP31 reporter (Figures 3E,F). The overexpression of miR-362-3p downregulated BCAP31 protein levels in HeLa and SiHa cells (Figure 3G). In cervical cancer tissues, miR-362-3p was oppositely associated with BCAP31 expression (Figure 3H).

Overall, these results indicate that miR-362-3p directly targets BCAP31, suggesting that miR-362-3p may exert its effect by inhibiting BCAP31 in cervical cancer.

### Down-Regulated BCAP31 Inhibits Migration and Invasion of Cervical Cancer Cells

To study the role of BCAP31 in cervical cancer metastasis, we first analyzed the correlation between the expression level of BCAP31 and the clinical-pathological parameters of patients. We collected the clinical samples for immunohistochemical staining and then quantified. The histochemical-scores were used to represent the protein expression level of BCAP31. The results showed that BCAP31 was correlated with cervical cancer lymph node metastasis and grade (Table 2).

**Table 2:** Correlation between expression of BAP31 in cancer cell and clinicopathological parameters.

**Factor**	**Low BAP31**		**High BAP31**		***P*-value**
	**No**.	**%**	**No**.	**%**	
All patients	104	50.0	104	50.0	
Age (years)					
≤ 40	23	11.1	22	10.6	
>40	81	38.9	82	39.4	0.866
Tumor size					
T1	68	32.7	64	30.8	
T2 or T3	36	17.3	40	19.2	0.565
Lymph node					
N0	103	49.5	96	46.2	
N1	1	0.5	8	3.8	**0.017**
Grade					
–	9	4.3	5	2.4	
1	26	12.5	5	2.4	
2 or 3	69	33.2	94	45.2	**<0.0001**
Stage					
I or II	101	48.6	96	46.2	
III or IV	3	1.4	8	3.8	0.121

*No. means the number of cases. Bold values indicates that the p < 0.05, which represents a statistical difference*.

**Figure 4:**
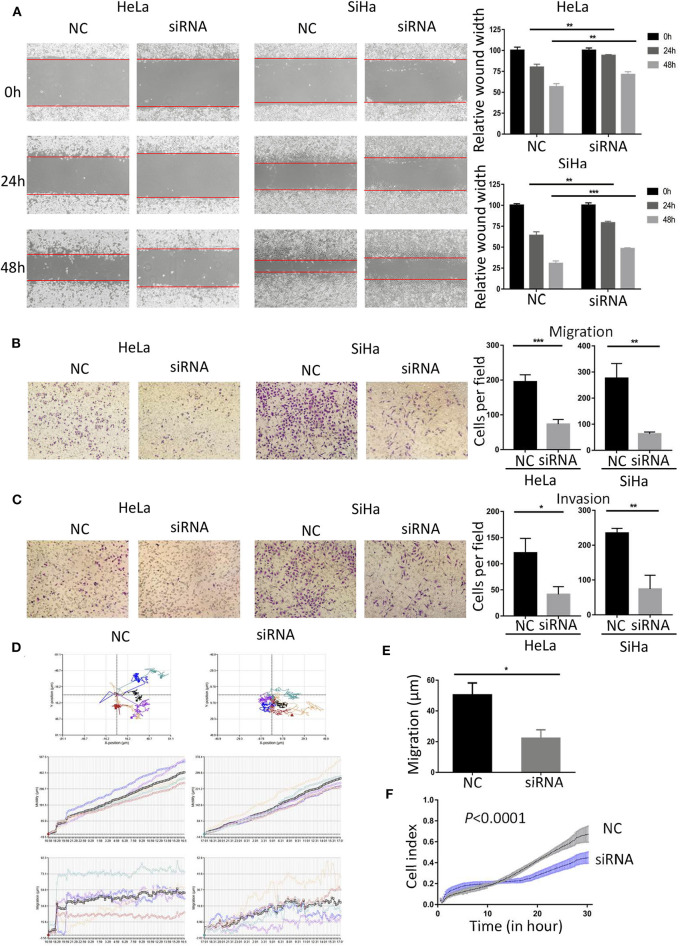
Down-regulated BCAP31 inhibits migration and invasion of cervical cancer cells. **(A)** Wound-healing experiments showed the migration of HeLa and SiHa cells transfected with negative control (NC) or BCAP31 siRNA. **(B)** Transwell migration assay of HeLa and SiHa cells after transfected with NC or BCAP31 siRNA. **(C)** Transwell invasion assay of HeLa and SiHa cells treated as in **(B)**. **(D)** HeLa cell movement tracks were recorded by HoloMonitor M4 to show cell migration ability, after transfected with NC or BCAP31 siRNA. **(E)** Cell migration distance results from five cells randomly selected in different directions after transfected with NC or BCAP31 siRNA in HeLa cells, tested by HoloMonitor M4. **(F)** Real-time cell migration was measured using the xCELLigence RTCA DP system, for HeLa cells transfected with NC or BCAP31 siRNA. ^**^P < 0.01; ^***^P < 0.001. Datas are represented as the mean ± s.d. of three independent experiments.

As the expression level of BCAP31 is related to the tumor metastasis of patients with cervical cancer, we studied the changes in cell migration and invasion ability after BCAP31 siRNA transfection at HeLa and SiHa cells. The results showed that the scratch healing ability of cells was significantly reduced after transfection, compared with the negative control group (Figure 4A). The results of transwell experiments proved that cell migration and invasion ability were both reduced after knockout BCAP31 (Figures 4B,C) without significant difference in cell counts between the two groups after mitomycin C treatment (Supplementary Figure S2). Real-time monitoring of the cell trajectory by HoloMonitor M4 showed that the cell's ability to migrate was decreased (Figures 4D,E). Consistently, the RTCA results also suggested that the number of migrating cells was reduced after transfection (Figure 4F).

The above results show that knockout BCAP31 can effectively reduce the ability of cervical cancer cells to migrate and invade. MiR-362-3p may regulate cervical cancer metastasis by targeting BCAP31.

### Enrichment Plots From Gene Set Enrichment Analysis

**Figure 5:**
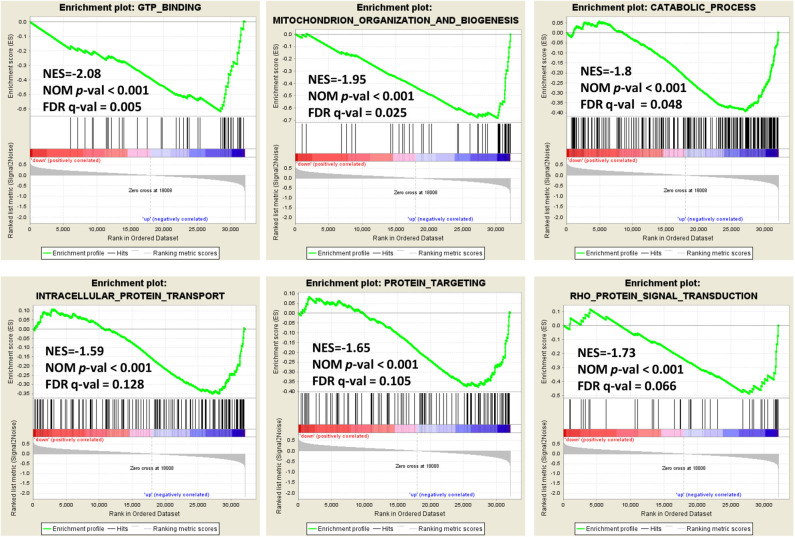
Enrichment plots from gene set enrichment analysis. Genome-wide data were downloaded from TCGA for available cervical cancer tumors. Several pathways and biological processes, including GTP binding, mitochondrion organization and biogenesis, catabolic process, intracellular protein transport, protein targeting and Rho protein signal transduction, were differentially enriched in cervical cancer cases with high and low BCAP31 expression.

In order to explore the role of BCAP31 in cervical cancer migration, we performed the Gene Set Enrichment Analysis (GSEA) on expression data from the TCGA database. Details of the top 30 enriched gene sets are provided in Supplementary Table S1. Gene sets related to GTP binding, mitochondrion organization and biogenesis, catabolic process, intracellular protein transport, protein targeting and Rho protein signal transduction, were differentially enriched in the BCAP31 expression phenotype (Figure 5).

## Discussion

Although the HPV vaccine application brings some good news, the incidence and mortality of cervical cancer remain high. Recent research shows that more than 500,000 women are diagnosed with cervical cancer each year, 85% of which occur in low-middle developed areas, and the disease causes more than 300,000 deaths worldwide (Vaccarella et al., 2013; Cohen et al., 2019). Metastasis and recurrence are leading causes of death in patients with cervical cancer. With the deepening of miRNA research, more and more studies have found that miRNAs are involved in the regulation of growth and metastasis of various tumors, including cervical cancer (Rupaimoole et al., 2016; Granados-Lopez et al., 2017; Mihaylova et al., 2019; Xu et al., 2019).

MiR-362 is a non-coding RNA consisting of 22 bases. Researchers have reported that miR-362 is abnormally expressed in breast cancer, renal cancer, gastric cancer and hepatocellular carcinoma (Shen et al., 2015; Zhang et al., 2015; Zou et al., 2016; Assiri et al., 2019). In colorectal cancer, researchers found that miR-362 may be involved in the regulation of the tumor cell cycle by affecting the expression of E2F1, USF2, and PTPN1 (Christensen et al., 2013). Some recent studies have found that miR-362 acts as a tumor suppressor miRNA for cervical cancer by targeting MCM5 and other potential targets (Wang et al., 2018; Song et al., 2019). This study demonstrated that miR-362-3p is abnormally low-expressed in cervical cancer, which is related to clinical clinicopathological parameters and patient survival. Cell experiments confirmed that miR-362-3p can effectively inhibit the migration and invasion of cervical cancer cells. MiR-362-3p can inhibit BCAP31 protein expression by directly targeting BCAP31 3′UTR.

BCAP31 is located on the endoplasmic reticulum membrane and is mainly involved in protein transport and apoptosis (Annaert et al., 1997; Nguyen et al., 2000; Paquet et al., 2004; Zen et al., 2004; Abe et al., 2009; Bartee et al., 2010; Iwasawa et al., 2011). In recent years, studies have found that BCAP31 is a novel tumor/testis antigen, which is abnormally highly expressed in many tumors, especially in cervical cancer (Yu et al., 2015; Dang et al., 2018; Wang et al., 2019). Our study in this article found that the knockout of BCAP31 reduced the ability of cervical cancer cells to migrate and invade. Further GSEA analysis showed that BCAP31 may participate in multiple biological processes, such as protein transport, metabolism and organelle organization. We have found in previous studies that BCAP31 may regulate the migration and invasion ability of cancer cells by regulating the expression of cytoskeletal proteins, such as Drebrin, M-RIP, SPECC1, Nexilin, and F-actin (Dang et al., 2018; Wang et al., 2020). These results are consistent with our GSEA analysis results. In this article, we also found that BCAP31 may participate in the regulation of tumor cell migration and invasion through GTP metabolism and RHO signal pathway. Given that BCAP31 is a transporter protein and participates in the ER-associated degradation pathway, we suspect that BCAP31 may transport and regulate the expression of multiple metastasis-related proteins. This may be the reason why BCAP31 regulates cell migration and metastasis.

There are some limitations in the present study. Although we have shown that miR-362-3p can regulate the migration and invasion of cervical cancer cells, it is unclear whether these cell phenotypic changes are caused only by changes in BCAP31 expression. The regulation effect of miR-362-3p mimics transfection group on cell metastasis is not weaker than the BCAP31 siRNA transfection group. It can be inferred that miR-362-3p may also function through other proteins and pathways. Other targeting molecules regulated by miR-362-3p need to be further researched. In clinical sample studies, we analyzed the correlation between miR-362-3p and BCAP31 expression and clinical-pathological parameters of cervical cancer patients. However, due to the limited number of clinical samples, we were unable to get samples with distant metastases, and the number of patients with lymph node metastases was also limited. This affected the results of statistical analysis to some extent. The larger sample size would be better. In addition, it is also necessary to use mouse models to further evaluate the potential of miR-362-3p for miRNA therapeutics.

In summary, our study shows that miR-362-3p plays an important role in the metastasis of CC and is related to the prognosis of patients. MiR-362-3p regulates the migration and invasion of cervical cancer cells by directly targeting BCAP31. MiR-362-3p may be a potential target for cervical cancer treatment.

## Data Availability Statement

All datasets generated for this study are included in the article/Supplementary Material.

## Ethics Statement

The studies involving human participants were reviewed and approved by Medical Ethics Committee of FMMU. The patients/participants provided their written informed consent to participate in this study.

## Author Contributions

KY and LZ designed the study. SY, XZ, and YS finished the experiments. SY, JS, DJ, and JW wrote the manuscript. YL, CH, and JP performed the statistical analysis of the data. All authors reviewed the manuscript.

## Conflict of Interest

The authors declare that the research was conducted in the absence of any commercial or financial relationships that could be construed as a potential conflict of interest.
