# Supplementary material for: MicroRNA-362-3p Inhibits Migration and Invasion via Targeting BCAP31 in Cervical Cancer
Source: Front Mol Biosci. 2020 Jun 9;7:107. doi: 10.3389/fmolb.2020.00107 (PMC7296163; doi:10.3389/fmolb.2020.00107)
Supplement: Supplementary file 1 [file Data_Sheet_1.pdf]

Supplementary Figures and Table

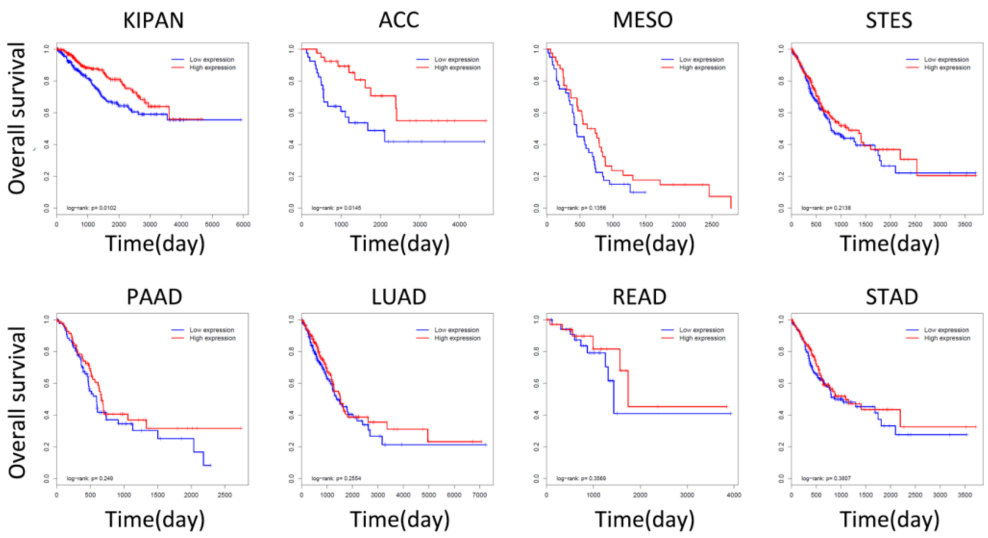

Supplementary Figure S1. Survival curves of different cancers with low/high miR-362 expression from TCGA database.

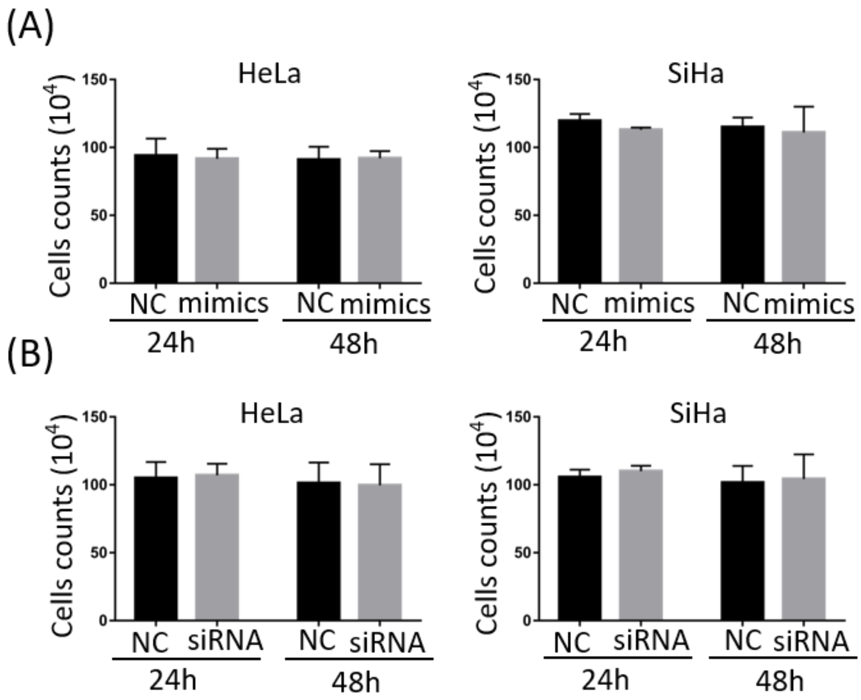

Supplementary Figure S2. There was no significant difference in the number of cells in each group after mitomycin C treatment.

(A) Cells counts at 24h and 48h after mitomycin C treatment in NC and miR-362-3p mimics groups. (B) Cells counts at 24h and 48h after mitomycin C treatment in NC and BCAP31 siRNA groups.

Supplementary Table S1. The top 30 enriched gene sets in the BCAP31 expression phenotype of cervical cancer data from TCGA

Gene set name	NES	NOM p-val	FDR q-val
PROTEIN_FOLDING	-2.28	<0.001	<0.001
RESPONSE_TO_OXIDATIVE_STRESS	-2.26	<0.001	<0.001
MITOCHONDRIAL_PART	-2.23	<0.001	<0.001
MITOCHONDRIAL_ENVELOPE	-2.19	<0.001	<0.001
MITOCHONDRION	-2.19	<0.001	<0.001
MITOCHONDRIAL_MEMBRANE_PART	-2.14	<0.001	0.002
ENVELOPE	-2.13	<0.001	0.004
ORGANELLE_ENVELOPE	-2.13	<0.001	0.004
MITOCHONDRIAL_MEMBRANE	-2.13	<0.001	0.003
GTP_BINDING	-2.08	<0.001	0.005
PROTEASOME_COMPLEX	-2.06	<0.001	0.006
GUANYL_NUCLEOTIDE_BINDING	-2.06	<0.001	0.007
ORGANELLE_MEMBRANE	-2.06	<0.001	0.006
HYDROGEN_ION_TRANSMEMBRANE_TRANSPORTER_ACTIVITY	-2.05	<0.001	0.007
UNFOLDED_PROTEIN_BINDING	-2.05	<0.001	0.006
ELECTRON_CARRIER_ACTIVITY	-2.04	<0.001	0.01
MITOCHONDRIAL_INNER_MEMBRANE	-2.03	<0.001	0.01
RIBOSOME	-2.03	<0.001	0.01
PHOSPHOTRANSFERASE_ACTIVITY_PHOSPHATE_GROUP_AS_ACCEPTOR	-2.02	<0.001	0.01
RIBONUCLEOPROTEIN_COMPLEX	-1.98	0.02	0.023
MACROMOLECULE_CATABOLIC_PROCESS	-1.97	<0.001	0.023
MITOCHONDRION_ORGANIZATION_AND_BIOGENESIS	-1.95	<0.001	0.025

MITOCHONDRIAL_RESPIRATORY_CHAIN	-1.95	<0.001	0.024
INDUCTION_OF_APOPTOSIS_BY_INTRACELLULAR_SIGNALS	-1.94	<0.001	0.025
ORGANELLE_INNER_MEMBRANE	-1.94	0.02	0.025
INDUCTION_OF_APOPTOSIS_BY_EXTRACELLULAR_SIGNALS	-1.93	<0.001	0.026
NEGATIVE_REGULATION_OF_TRANSCRIPTION_FACTOR_ACTIVITY	-1.92	<0.001	0.028
MONOVALENT_INORGANIC_CATION_TRANSMEMBRANE_TRANSPORTER_ACTIVITY	-1.92	<0.001	0.028
NUCLEOBASENUCLEOSIDENUCLEOTIDE_KINASE_ACTIVITY	-1.92	<0.001	0.026
RIBONUCLEASE_ACTIVITY	-1.92	0.02	0.027

NES: normalized enrichment score; NOM: nominal; FDR: false discovery rate. Gene sets with NOM P-values <0.05 and FDR q-values <0.25 were considered significantly enriched.
